# Profile of Mood States Factor Structure Does Not Accurately Account for Patients with Chronic Pain

**DOI:** 10.1093/pm/pnab127

**Published:** 2021-04-02

**Authors:** Celia María López-Jiménez, Francisco Javier Cano-García, Susana Sanduvete-Chaves, Salvador Chacón-Moscoso

**Affiliations:** Departamento de Personalidad, Evaluación y Tratamiento Psicológicos, Universidad de Sevilla, Seville, Spain; Departamento de Personalidad, Evaluación y Tratamiento Psicológicos, Universidad de Sevilla, Seville, Spain; Departamento de Psicología Experimental, Facultad de Psicología, Universidad de Sevilla, Seville, Spain; Departamento de Psicología Experimental, Facultad de Psicología, Universidad de Sevilla, Seville, Spain; Universidad Autónoma de Chile, Santiago, Chile

**Keywords:** Chronic Pain, Initiative on Methods, Measurement, and Pain Assessment in Clinical Trials (IMMPACT), Profile of Mood States (POMS), Confirmatory Factor Analysis, Exploratory Factor Analysis

## Abstract

**Objective:**

The need for measuring emotional functioning in patients with chronic pain was recognized decades ago. The Initiative on Methods, Measures, and Pain Assessment in Clinical Trials (IMMPACT) proposed the Profile of Mood States for this purpose. However, to date, its factor structure has not been confirmed in these patients.

**Methods:**

We set out to use confirmatory factor analysis to test the theoretical structure of seven factors: Tension-Anxiety, Depression-Dejection, Anger-Hostility, Vigor-Activity, Fatigue-Inertia, Confusion-Bewilderment, and Friendliness.

**Participants:**

The sample consisted of 588 Spanish adult patients with chronic pain.

**Results:**

The original structure could not be verified according to the obtained fit indices (e.g., root-mean-square error of approximation = 0.11). For this reason, we carried out a second study that relied on exploratory factor analysis to evaluate the structure in half of the cases and confirmatory factor analysis to validate it in the other half. The factor structure detected in the exploratory factor analysis was not satisfactory, nor could it be validated with confirmatory factor analysis (e.g., normed fit index between 0.54 and 0.56).

**Conclusions:**

The factor structure of the Profile of Mood States could not be satisfactorily confirmed. Consequently, other mood measures and shorter, optimized versions of the POMS are discussed as possible alternatives.

## Introduction

Chronic pain is a public health problem [1]. Scientific literature has shown the complex and multifactorial nature of pain [2, 3]. An adequate approach to pain requires that all of its dimensions be considered, from sensation to meaning [4].

The Initiative on Methods, Measurement, and Pain Assessment in Clinical Trials (IMMPACT) [5], comprising specialists from academia, government agencies, and the pharmaceutical industry, was introduced in order to improve the overall quality of pain assessment in research. IMMPACT’s initial aims were to propose core and supplemental domains of pain assessment [6], as well as to select, improve, and promote measures for each proposed assessment domain [7]. One of the core domains proposed under IMMPACT was emotional functioning, and the main measure chosen to evaluate it, in addition to the Beck Depression Inventory [8], was the Profile of Mood States (POMS) [9–11].

The POMS was designed to measure affective states in psychiatric patients [12]. It consists of 65 adjectives describing feelings grouped in six mood dimensions: Tension-Anxiety, Depression-Dejection, Anger-Hostility, Vigor-Activity, Fatigue-Inertia, and Confusion-Bewilderment. The items constituting Friendliness were excluded because of poor discriminant validity with the Vigor-Activity scale. From the remaining 58 items, a global score on total mood disturbance can be obtained by calculating the sum of the scores, except for Vigor-Activity, which is subtracted. The original study achieved good psychometric properties in samples of outpatient drug trials, psychotherapy, or a combination of psychotherapy and drug treatments: internal consistency (alphas) from 0.84 to 0.95, test–retest reliability from 0.65 to 0.74, and concurrent validity with Minnesota Multiphasic Personality Inventory scales from –0.58 to 0.69.

Many studies using the POMS have provided cross-cultural validation, in languages including German [13], Dutch [14], Spanish [15], French [16], Korean [17], Portuguese [18], Chinese [19], and Hebrew [20], among others. The POMS has been used with a wide variety of samples: general population [21], athletes [22], delinquents [23], psychiatric patients [24], and postmenopausal women [25], and among patients suffering from diseases such as metabolic syndrome [26], multiple sclerosis [27], HIV [28], encephalomyelitis [29], and especially cancer [30].

With the exception of the studies on shortened versions of the POMS [31, 32] or a recent second version of the full test [33], its structural validity has received little attention. The original factor structure has been confirmed in undergraduate students [15, 34], prison inmates [34], postmenopausal women [25], patients addicted to cocaine [35], older adults [36], and athletes [15, 34, 37]. However, other studies were unable to confirm certain factors—for example, Confusion-Bewilderment in athletes and undergraduate students [38] or in older adults [39], and Tension-Anxiety, Depression-Dejection, and Confusion-Bewilderment in psychiatric outpatients and adult smokers [40]. Finally, other authors report structures with more than six factors in undergraduate students [41].

The POMS was chosen by the IMMPACT Initiative because it is brief, easy to administer, and useful for nonpsychiatric populations, and it considers a variety of mood states, including anxiety, depression, and anger, the most prevalent symptoms among pain patients [9].

In chronic pain research, and despite the IMMPACT recommendations, the POMS has been used only infrequently; as far as we know, only seven studies have used it to date [42–48], usually for testing the efficacy of treatments. However, none of them tested the original factor structure. Consequently, our aim was to study the factor structure of the POMS in patients with chronic pain.

## Methods

### Participants

The sample comprised 588 patients with chronic pain from the province of Seville (Andalusia, Spain). Of the 588 patients, 432 had been selected to participate in a group psychoeducational program provided at eight different primary care centers, 75 had been assigned to a group self-hypnosis program provided at a fibromyalgia association, 41 were going to attend a seminar for patients with chronic pain at a community center, and 40 were to receive an individual cognitive restructuring intervention at another primary care center. The sociodemographic characteristics and clinical data of the pooled sample are shown in Tables 1 and 2, respectively.

**Table 1.** Sociodemographic characteristics of the sample (n = 588)

	Mean (SD)	Range
	n	%
Age, years	54 (9.7)	29–87
Gender		
Female	538	91.5
Male	50	8.5
Race/ethnicity		
White/Caucasian	588	100
Employment		
Housewife	209	35.5
Employed	130	22.1
Unemployed	98	16.7
Permanent disability	75	12.8
Retired	47	8
Temporary disability	28	4.8
Unknown	1	0.1
Education		
Primary	302	51.4
Secondary	150	25.5
No schooling	83	14.1
Higher	52	8.8
Unknown	1	0.2
Marital status		
Married	453	77
Separated/divorced	56	9.5
Widowed	42	7.1
Single	35	6
Unknown	2	0.4

**Table 2.** Clinical data of the sample (n = 588)

	Mean(SD)	Range
	n	%
Pain chronicity (in years)	14.9 (9.7)	1–60
Pain intensity (last 24 hours)*	7.1 (2.3)	0–10
Pain intensity (during assessment)*	5.7 (2.8)	0–10
Pain duration (in hours)^†^	19.9 (7.3)	1–48
Diagnosis		
Fibromyalgia	347	59
Arthrosis	57	9.7
Chronic lower back pain	51	8.7
Other osteoarticular disease	45	7.6
Chronic headache	35	6
Unknown diagnosis	21	3.6
Herniated disc	16	2.7
Chronic cervicalgia	8	1.4
Rheumatoid arthritis	7	1.2
Neuropathic pain	1	0.1
Diagnosed by		
Rheumatologist	195	33.2
Traumatologist	177	30.1
Family doctor	148	25.2
Unknown	32	5.4
Neurologist	24	4.1
Pain doctor	9	1.5
Internist	3	0.5
Pain interference (last 24 hours)		
Moderate (hampers daily activity)	340	57.8
Severe (prevents daily activity)	166	28.2
Slight (does not hamper or prevent)	51	8.7
None	29	4.9
Unknown	2	0.3
Pain interference (during assessment)		
Moderate (hampers daily activity)	285	48.5
Slight (does not hamper or prevent)	123	20.9
Severe (prevents daily activity)	102	17.3
None	74	12.6
Unknown	4	0.7
Pain frequency		
Daily	494	84
Day in and day out	37	6.3
Twice a week	27	4.6
Four times a week	11	1.9
Twice a month	11	1.9
Unknown	8	1.4

* Numerical Rating Scale (NRS).

† Duration of each episode or pain crisis. People with chronic pain responded “24 hours a day.”

### Measures

The Spanish version of the POMS [15] was used to gather the data (see Supplementary Data File 1). The verification of the translation was done with a back-translation, in keeping with the International Test Commission recommendations [49]. Each item is an adjective that participants must rank on a five-point Likert scale. In the English version, the instructions are as follows: “Read each word/statement below, decide how you have been feeling, in respect to the word/statement, in the past week and today, and select the appropriate statement ‘Not at All,’ ‘A Little,’ ‘Moderately,’ ‘Quite a Lot’ or ‘Extremely’ to indicate your feeling.”

The original 65 items were grouped into seven factors as follows: Tension-Anxiety, nine items (tense, shaky, on edge, panicky, relaxed, uneasy, restless, nervous, anxious); Depression-Dejection, 15 items (unhappy, sorry about things done, sad, blue, hopeless, unworthy, discouraged, lonely, miserable, gloomy, desperate, helpless, worthless, terrified, guilty); Anger-Hostility, 12 items (angry, peeved, grouchy, spiteful, annoyed, resentful, bitter, ready to fight, rebellious, deceived, furious, bad tempered); Vigor-Activity, eight items (lively, active, energetic, cheerful, alert, full of life, carefree, vigorous); Fatigue-Inertia, seven items (worn out, listless, fatigued, exhausted, sluggish, bushed, weary); Confusion-Bewilderment, seven items (confused, unable to concentrate, muddled, bewildered, efficient, forgetful, uncertain about things); and Friendliness, seven items (friendly, clear headed, considerate, sympathetic, helpful, good natured, trusting).

The total score for a participant is obtained by inverting the values of the *relaxed* items (the Tension-Hostility factor) and *efficient* items (the Confusion-Bewilderment factor) and calculating the sum of all items except the Vigor-Activity factor, which is subtracted. Scores oscillate from –28 to 232. A high score indicates a negative mood state, and vice versa.

Other measures recommended by the IMMPACT initiative [7] were obtained (see Table 2): pain chronicity, pain intensity, pain duration, pain interference, and pain frequency.

### Procedures

This study was carried out in accordance with the recommendations of the Ethics Committee of the Southern Seville Health District (Andalusian Health Service). All participants gave written informed consent in accordance with the Declaration of Helsinki.

The inclusion criteria were the following: 1) age 18 years or older; 2) to have a chronic pain diagnosis by a Spanish Health System practitioner; 3) to have visited primary care because of difficulties handling chronic pain during the recruitment period (present maladaptive adjustment to pain); 4) to not be in the middle of an employment dispute or waiting for approval on a disability pension; 5) to not have a primary psychopathological disorder; 6) to not be in psychiatric or psychological treatment, but could be taking analgesic, anxiolytic, or antidepressant drugs; 7) to be able to follow group sessions, thus excluding conditions such as deafness, blindness, or dementia; 8) willing to sign an agreement to attend the sessions (group and/or individual); and 9) to not be hospitalized. With regard to criterion number 3, all patients who stated that chronic pain was their main problem were included (e.g., patients also suffering from anxiety or depression who attributed this disorder to the chronic pain). The 41 participants who were going to attend a seminar for patients with chronic pain were required to meet all of the criteria except number 3.

In each study, groups of eight to ten people collectively self-administered the measures before the planned activity or intervention under the supervision of and with the support of two graduate students in clinical psychology. Participants who received a psychological intervention completed the same battery of tests at the end (post-test) and after 6–9 months (follow-up). The POMS data used in the present article corresponded to the baseline measure (pre-test).

### Study 1

In this study, we tested the complete original factor structure (all 65 items) by using confirmatory factor analysis (CFA) in Model 1. Model 2 is a CFA test without the Friendliness factor (58 remaining items), given that this factor was struck from the original version for psychometric reasons [12].

### Data Analysis

SPSS 25 (IBM Corporation, Armonk, NY, USA) was used to store the data and calculate the reliability coefficient (internal consistency) and the average discrimination index, both of which were considered essential requirements, before proceeding to analyze the factor structure.

The reliability coefficient was calculated with Cronbach’s alpha. Values >0.90 were considered excellent; 0.80–0.90, good; 0.70–0.80, acceptable; 0.60–0.70, questionable; 0.50–0.60, poor; and <0.50, unacceptable [50, 51]. The average discrimination index was interpreted as excellent for values >0.40, good for values of 0.30–0.40, adequate for 0.20–0.30, and inadequate for values <0.20 [52].

PRELIS and LISREL 9.3 (SSI Inc, Chapel Hill, NC, USA) were used to estimate the polychoric correlation matrix [53, 54], verify the bivariate normal distribution (a necessary assumption to be able to use polychoric correlations), and carry out the CFA. Pairwise deletion was used for missing data. The assumption of bivariate normal distribution was checked with the chi-squared test (χ^*2*^) and the percentage of tests that rejected the null hypothesis of bivariate normality for each pair of correlations. A 95% confidence level was assumed, and the Bonferroni correction was applied, with the following formula used to calculate the value of *α* to use in comparing each contrast: *α*/c [*α* = 0.05 corresponding to a 95% confidence level; c = the number of contrasts = (number of items × number of items – 1)] / 2. Because of the sensitivity of χ^*2*^ when the sample size is large, we also calculated the root-mean-square error of approximation (RMSEA), where values that did not exceed 0.1 would imply no significant effect on the parameter estimate [55].

The original factor structure was tested with CFA, as was the same structure without the Friendliness factor [56]. The two tested models were second-order factor models that measured mood state, formed by seven and six factors, respectively. Unweighted least squares were used for the estimates, as these are appropriate for polychoric correlations and ordinal variables with asymmetrical distribution [54, 57]. As the correlation matrix between factors was not specified, the program calculated it as symmetrical and free by default, thus correlating all the second-order factors. The lambda parameter corresponding to the relationship of the first item with each factor was set to one to solve the model identification problem and determine the measurement scale of the latent variables.

Next, the standardized factor loadings were calculated. Lambdas over 0.30 were considered acceptable, regardless of whether they were positive or negative. Additionally, several fit indices were used to assess the adequacy of the models: 1) the χ^*2*^ test, where *P* > 0.05 meant a good fit; 2) the expected cross-validation index (ECVI), with acceptable model fitness when the value of the index was closer to that of the saturated model than of the independence model (the lower the value, the better the fit) [58]; 3) the RMSEA [55], where values less than 0.05 were considered a good fit, values between 0.08 and 0.10 a reasonable fit, and values greater than 0.10 unfit [59]; as well as 4) the goodness-of-fit index (GFI); 5) the adjusted goodness-of-fit index (AGFI) [55]; 6) the comparative fit index (CFI) [60]; 7) the normed fit index (NFI); and 8) the non-normed fit index (NNFI) [61], where values above 0.90 indicated a good fit [62].

## Results

###  

#### Reliability

Factors presented reliability coefficients that ranged from acceptable to excellent results: in Tension-Anxiety, *α* = 0.88 (good); in Depression-Dejection, *α* = 0.91 (excellent); in Anger-Hostility, *α* = 0.90 (good, close to excellent); in Vigor-Activity, *α* = 0.75 (acceptable); in Fatigue-Inertia, *α* = 0.83 (good); in Confusion-Bewilderment, *α* = 0.76 (acceptable); and in Friendliness, *α* = 0.74 (acceptable). The global reliability coefficient was excellent in both Model 1 (considering the 65 items) and Model 2 (removing the Friendliness factor), with *α* = 0.95 and 0.96, respectively.

#### Discrimination Index

This index yielded good results for the following factors: Tension-Anxiety (*D* = 0.61), Depression-Dejection (*D* = 0.59), Anger-Hostility (*D* = 0.57), Fatigue-Inertia (*D* = 0.57), and Confusion-Bewilderment (*D* = 0.49). Although Vigor-Activity yielded an adequate result (*D* = 0.24), the result for Friendliness was inadequate (*D* = –0.02). As can be seen in Table 3, the global average discrimination index was good in Model 1 and better in Model 2 (omitting Friendliness). Although these results indicate that the Friendliness factor did not work properly, we continued testing the factor structure of both Models 1 and 2, supposing that Model 1 would not fit.

**Table 3.** Summary of confirmatory factor analyses (CFA) in Studies 1 and 2

Study	Model	α	*D*	χ^2^(df)	ECVI (Saturated–Independence)	RMSEA	GFI	AGFI	CFI	NFI	NNFI
1	1	**0.951**	**0.457**	14965.849(2009)*	**25.915 (7.296–54.905)**	0.105	**0.925**	**0.919**	0.569	0.535	0.554
1	2	**0.959**	**0.515**	15209.807(2017)*	**26.302 (7.296–54.905)**	0.105	**0.913**	**0.907**	0.561	0.527	0.548
2	3	**0.959**	**0.514**	6147.348(1646)*	**21.753 (12.041–45.36)**	**0.0964**	**0.940**	**0.936**	0.609	0.535	0.593
2	4	**0.960**	**0.529**	5652.994(1531)*	**20.058 (11.245–44.146)**	**0.0957**	**0.953**	**0.949**	0.634	0.561	0.619

Model 1 (65 items)= complete original factor structure; Model 2 (58 items) = original factor structure omitting the Friendliness factor. Model 3 (59 items) = CFA based on the EFA with the full version of the test. Model 4 (57 items)= CFA based on the EFA after removing the Friendliness factor. Adequate results are marked in bold.

* *P* < 0.001.

#### Bivariate Normal Distribution

Given that 65 items were included in Model 1, a total of 2,080 correlations were obtained (65 × 64/2). Based on χ^2^, the bivariate normality assumption was met on 79.9% of the occasions (1,661 correlations), comparing their significance with *P* = 0.05 / 2080 = 0.00002 after applying the Bonferroni correction. Moreover, the RMSEA was less than 0.1 on 99.9% of occasions (2,079 correlations).

In Model 2, after omission of the seven items that formed the Friendliness factor, 58 items were considered, yielding 1,653 bivariate correlations (58 × 57/2). χ^2^ obtained a *P* > 0.00003 (Bonferroni correction: 0.05 / 1653) on 82.8% of the occasions (1,368 correlations). Furthermore, the RMSEA was less than 0.10 on 99.8% of occasions (1,650 correlations). Once it was verified that these results allowed the matrix of polychoric correlations to be used in both Models 1 and 2, the CFAs were carried out.

#### CFA

Table 3 shows that the original structure was not confirmed in either of the two models. In Model 1, some indices yielded acceptable results: ECVI was closer to the saturated model than to the independence model; GFI and AGFI were >0.90. Nevertheless, the rest of the fit indices were poor. Lambdas ranged from 0 to 0.93 (all lambdas are listed in Supplementary Data File 2). Only three items presented *λ* < 0.30. Gammas ranged from 0.02 (Friendliness) to 0.98 (Depression-Dejection), five of them being over 0.87.

The fit of Model 2 was slightly worse than that of Model 1. Appropriate results were found in ECVI, with a value closer to the saturated model than to the independence model; GFI and AGFI were >0.90. However, the results of the rest of the indices indicated a poor fit. Lambdas ranged from 0.28 to 0.86 (all lambdas are listed in Supplementary Data File 2). Only two items presented *λ* < 0.30; with regard to the gammas, all were over 0.88 except the one related to Vigor-Activity, which obtained *γ* = –0.58.

### Study 2

Given the deficient fit indices obtained in both models in Study 1, we wished to determine whether there was an alternative to the original structure that fit with our sample without altering the substantive meaning. For that, we carried out one exploratory factor analysis (EFA) with all 65 items (Model 3) and a second EFA omitting the seven items that comprise the Friendliness factor (Model 4). Finally, we conducted the corresponding CFA to corroborate the structure obtained in the EFAs.

#### Data Analysis

The same database used in Study 1 was randomly divided into two equal parts, each with 294 participants. The first half was used for the EFA, and the second half served to check the structure obtained in the EFA by using the CFA. For missing data throughout the analysis, pairwise deletion was used.

To perform the EFA, two polychoric correlation matrixes were used: one consisting of 65 items [53] and another of the 58 items remaining after the seven items of the Friendliness factor had been omitted. Before that analysis, we assessed whether that matrix presented the assumptions necessary to conduct an EFA, calculating the Kaiser-Meyer-Olkin (KMO) test (values over 0.70 would be considered acceptable) and Bartlett’s test of sphericity (statistically significant χ*^2^* would imply acceptable results) with SPSS 25. Once the corresponding assumptions had been checked, an EFA was performed in PRELIS and LISREL 9.3, with the MINRES factor analysis used as an estimation method (acceptable for ordinal variables) and the PROMAX method used for oblique rotation, as we assumed that the factors would be correlated. Factors with initial eigenvalues over one were considered as part of the first stage. Lambdas greater than or equal to 0.30 were considered acceptable, regardless of whether they were positive or negative. When *λ* was greater than or equal to 0.30 for an item in more than one factor, the option that more closely aligned with the original model and with the meaning of the other items in the same factor was chosen. Items that did not fit with the other items assigned to a concrete factor meaning-wise were eliminated from the CFA. Factors comprising zero items or one item after the items had been assigned based on alignment with the original model and with the meaning of the other items included in the same factor were eliminated. Finally, factors with two or three items without any common meanings to be clustered were also struck from the CFA.

The data analysis completed for the CFA was the same as that explained in Study 1. Additionally, modification indices were obtained in order to be able to optimize fitness while maintaining substantive meaning when necessary.

## Results

### EFA with the Full Version (65 Items)

With all original 65 items included, the KMO test yielded 0.92. Bartlett’s test of sphericity yielded χ^*2*^(2080) = 10993.176, *P* < 0.001. These results verified the assumptions necessary to carry out the EFAs.

Originally, the EFA presented 13 factors (see Supplementary Data File 3). Lambdas of 0.30 or higher were marked in bold. Considering the range of *λ* for each item, the lowest value was –0.30 (item 51, “alert,” in factor 6); the highest *λ* was 0.95 (item 17, “grouchy,” in factor 3). We obtained *λ* ≥ 0.30 for 40 items (61.5%) in one factor (items 4, 6, 8, 11, 13, 14, 15, 16, 17, 19, 21, 24, 25, 26, 27, 29, 30, 31, 32, 34, 35, 37, 38, 39, 41, 42, 43, 44, 45, 47, 48, 51, 52, 55, 56, 60, 62, 63, 64, and 65). We obtained *λ* ≥ 0.30 for 20 items (30.8%) in two factors (items 1, 3, 5, 9, 12, 18, 20, 22, 23, 28, 33, 36, 40, 46, 49, 50, 53, 54, 58, and 61). We obtained *λ* ≥ 0.30 for the remaining five items (7.7%) in three factors (items 2, 7, 10, 57, and 59). At a glance, factor 4 seemed to be Vigor-Activity, and factor 9, Friendliness. Factor 13 presented items referring to Fatigue-Inertia. Factors 2, 3, and 7 showed negative affectivity, blending Tension-Anxiety, Depression-Dejection, Confusion-Bewilderment, Anger-Hostility, and Fatigue-Inertia. Factors 1 and 6 presented Anger-Hostility items. The remaining factors (5, 8, 10, 11, and 12) seemed to be irrelevant. The EFA did not produce any clear factor solution. The values ultimately chosen for testing in the CFA are underlined in Supplementary Data File 3. Factor 2, formed by two items, was understood as Confusion-Bewilderment. Factor 3, formed by 27 items, was labeled Tension/Anger/Sadness (negative affectivity). Factor 4, formed by 10 items, was considered Vigor-Activity. Factor 6, formed by three items (spiteful, resentful, and alert), seemed to basically refer to Resentment, which is slightly different from Anger, although the negative connotation of the “alert” item, originally included in “Vigor,” casts doubt on its interpretation. Factor 7, formed by 11 items, was identified as Depression-Dejection. Factor 9, with four items, was identified as Friendliness. Factor 13, with two items, was identified as Fatigue-Inertia. In short, seven factors were included in the CFA; factors 1, 5, 8, 10, 11, and 12 (crossed out in Supplementary Data File 3) were not considered. Specifically, factor 1 presented five items with *λ* ≥ 0.30, and when four of them were reassigned to another factor because of a better fit meaning-wise and statistically (a higher *λ* in the factor to which it was reassigned), only one remained. For factor 5, only two items without any common meaning to allow a cluster (considerate and relaxed) fit. Factor 8 presented two items with *λ* ≥ 0.30, one of which (tense) was a better fit with factor 3 in both statistical terms and meaning-wise. For factor 10, after four items with *λ* ≥ 0.30 were reassigned to different factors because of their meaning, two items without any apparent meaning in common remained (rebellious and carefree). For factor 11, the only three items with *λ* ≥ 0.30 fit better with a different factor both statistically and meaning-wise. Finally, factor 12 uniquely obtained three items with *λ* ≥ 0.30, and all were reassigned to other factors because of a better fit statistically and meaning-wise. Moreover, six items (crossed out in Supplementary Data File 3) were removed from the CFA (clear-headed, considerate, relaxed, ready to fight, rebellious, and carefree) because they did not fit any of the factors either statistically (*λ* ≥ 0.30) or meaning-wise.

### CFA Based on the EFA with the Full Version (Model 3; 59 Items Remaining)

#### Internal Consistency

Internal consistency was excellent for the remaining 59 items (*α* = 0.96). Divided by factors, factor number 3, Tension/Anger/Sadness (negative affectivity), had an excellent result (*α* = 0.96); factors 4, Vigor-Activity, and 7, Depression-Dejection, had good results (*α* = 0.80 and 0.84, respectively); factor 13, Fatigue-Inertia, had acceptable results (*α* = 0.77); factors 2, Confusion-Bewilderment, and 6, Resentment, had questionable results (*α* = 0.62 and 0.64, respectively); and finally, factor 9, Friendliness, had a poor result (*α* = 0.60). Results below 0.70 could be influenced by the low quantity of items in certain factors (two items in factor 2, three items in factor 6, and four items in factor 9).

#### Average Discrimination Index

As can be seen in Table 3, the average discrimination index was excellent. Four of the factors yielded excellent results (factor 2, *D *= 0.58; factor 3, *D *= 0.65; factor 7, *D *= 0.53; and factor 13, *D *= 0.56). Factor 4 had a good result (*D *= 0.34). Factors 6 and 9 fell just short of acceptability (0.20 and 0.17, respectively). Despite the poor results in reliability and discrimination, we tested the corresponding factor structure.

#### Bivariate Normality Assumption

The results supported the use of polychoric correlations for the factor analysis. Specifically, with 59 items included, 1,711 bivariate correlations were calculated (59 × 58/2). When compared with *P* = 0.05 / 1711 = 0.000003 after application of the Bonferroni correction, the normality assumption based on χ^2^ was accepted on 95.4% of the occasions (1,633 correlations). Moreover, the RMSEA values were below 0.1 in 99.6% of the cases (concretely, 1,704 correlations).

#### Model Fit

The tested structure could not be confirmed (see Table 3). Some fit indices yielded adequate results. The ECVI for the saturated model was closer than the ECVI for the independence model (GFI and AGFI >0.90). The RMSEA also showed a reasonable fit. However, the rest of the fit indices were poor. Lambdas are available in Supplementary Data File 3. Lambdas ranged from 0.03 to 0.86. We obtained *λ* < 0.30 for five items, and gammas ranged from 0.36 (Friendliness) to 0.99 (Tension/Anger/Sadness), five of which were over 0.70.

### The EFA Without the Friendliness Factor (Maintaining 58 Items)

With the 58 items remaining after the seven items corresponding to the Friendliness factor were removed, the value of the KMO test was 0.93, and Bartlett’s test of sphericity yielded χ^*2*^(1653) = 9978.17, *P* < 0.001. Originally, 11 factors were found (see Supplementary Data File 3). Factor loadings over 0.30 are marked in bold. If we look at the highest *λ* in each item, the lowest value is –0.33 (item 51, “alert,” in factor 4), and the highest is 0.92 (item 17, “grouchy,” in factor 3). One item (1.7%) did not obtain *λ* ≥ 0.30 for any factor (item 23), so it was removed in the subsequent CFA. We obtained *λ* ≥ 0.30 for 36 items (62.1%) in one factor (items 3–19, 24, 28–32, 35, 37, 41, 46, 47, 51, 54, 56, and 58–65), *λ* ≥ 0.30 for 16 items (27.6%) in two factors (items 20–22, 26, 27, 34, 38, 39, 42–45, 48, 50, 52, 53, and 57), and *λ* ≥ 0.30 for five items (8.6%) in three factors (items 2, 33, 36, 40, and 49). The factor loadings ultimately chosen for inclusion in the following CFA are underlined in Supplementary Data File 3. The factor–item correspondence did not fit the original proposal [12]. Factor 2 contained items referring to Confusion-Bewilderment. Factor 9 seemed to be Vigor-Activity. Factor 11 included Fatigue items. Factor 3 was considered Tension-Depression-Anger. Factors 1 and 7 were considered irrelevant and thus excluded from the CFA. Specifically, factor 1 had only two items with *λ* > 0.30 (tense and miserable), and both were reassigned to other factors because of their meaning. In factor 7, the same occurred with the two items with *λ* > 0.30 (exhausted and ready to fight). The remaining factors were labeled on the basis of the substantive content of the items that saturated. Factor 4, formed by three items (spiteful, resentful, and alert), was labeled “Resentment.” Factor 5, formed by three items (restless, unable to concentrate, and nervous), was labeled “Nervousness.” Factor 6, formed by seven items (worn out, lonely, miserable, muddled, helpless, forgetful, and guilty), was labeled “Despondency.” Factor 8 was formed by four inverse items (hopeless, bewildered, deceived, and terrified) and was labeled “Determination” to account for the negative loading value of these items. Here, it is critical to consider the difficulty of interpreting items listed under three different factors in the original structure. Finally, Factor 10, formed by three items (two items with negative values—sluggish and rebellious–and one additional item, carefree), was labeled “Hedonism.” Again, the decision here can be attributed to the positive value and higher load of the final item, “carefree,” while again considering that these three items also corresponded to three different factors in the original structure. The resulting structure was tested in the CFA.

### The CFA Based on the EFA After Removal of the Friendliness Factor (Model 4; 57 Remaining Items)

#### Internal Consistency

The global reliability was excellent (*α* = 0.96). In terms of the factors, number 3, Tension-Depression-Anger (negative affectivity), yielded an excellent result (*α* = 0.94). Factor 6, Despondency, and factor 9, Vigor-Activity, yielded good results (*α* = 0.80 and 0.82, respectively). Factor 2, Confusion-Bewilderment, factor 5, Nervousness, factor 8, Determination, and factor 11, Fatigue, yielded acceptable results (*α* = 0.77, 0.72, 0.80, and 0.78, respectively). Factor 4, Resentment, yielded a questionable result (*α* = 0.64), and finally, factor 10, Hedonism, yielded an unacceptable result (*α* = 0.44).

#### Average Discrimination Index

The average discrimination index was excellent (see Table 3). By factors, all but one yielded excellent results (factor 2, *D *= 0.57; factor 3, *D *= 0.67; factor 4, *D *= 0.45; factor 5, *D *= 0.54; factor 6, *D *= 0.52; factor 8, *D *= 0.61; factor 9, *D *= 0.52; and factor 11, *D *= 0.62). Factor 10 yielded an adequate result (*D *= 0.27).

#### Bivariate Normality Assumption

With the 57 items, 1,596 bivariate correlations were calculated (57 × 56/2). The Bonferroni correction yielded *P* = 0.05 / 1596 = 0.00003. When this value was compared with the significances of χ^2^, the normality assumption was accepted on 95.9% of the occasions (1,530 correlations). Additionally, the RMSEA values were below 0.10 in 1,583 correlations (99.2% of the occasions). In short, the results supported the use of polychoric correlations.

#### Model Fit

As can be seen in Table 3, the tested structure was not confirmed. Some fit indices yielded acceptable results: The saturated model yielded a value closer to the ECVI than the independence model, a reasonable fit for the RMSEA, and a GFI and AGFI >0.90. Nevertheless, the remaining fit indices were poor. Lambdas are available in Supplementary Data File 3. Lambdas ranged from 0.23 to 0.84. Three items yielded *λ* < 0.30. Gammas ranged from 0.55 (Vigor-Activity) to 0.98 (Determination). Five gammas (77.8%) were over 0.70.

## Discussion

When Haythornthwaite and Edwards [10] proposed the POMS as the main measure of emotional functioning for clinical trials of pain at the IMMPACT-IV meeting, they relied on the psychometric information they had available (good internal consistency, test–retest reliability, and good convergent validity with other psychopathological measures). However, with regard to construct validity, they relied on the six-factor structure reported in various studies, none of which dealt with pain patients. Although the IMMPACT initiative ultimately adopted the proposal [9–11], no specific studies have been conducted in this regard. Therefore, our objective was to confirm the factor structure of POMS in patients with chronic pain.

We started by reviewing seven studies that had confirmed the original structure of six factors [15, 25, 35–37], although the number of factors had to be forced in one [34]; three others were not able to confirm any of the factors, not even when forcing six [38, 39] or seven factors [40]; and one study even obtained more than six [41]. None of these studies used pain patient samples.

In our chronic pain sample, it was not possible to confirm the original structure with six or seven factors. For this reason, and given that we had a sufficiently large sample, we carried out a second study in order to explore the structure with half of the cases and validate it with the other half. We were unable to find a satisfactory structure as reflected by the fit indices reported in the *Results* section, either when including the Friendliness factor items (the seven-factor model) or omitting them (the six-factor model). Previous studies have noted the poor performance of the Friendliness factor. Consequently, we conclude that the full version of the POMS is not appropriate for measuring the emotional functioning of patients with chronic pain and its implications.

The substantive analysis of the grouping of items in the EFA reveals a dispersion from the six or seven original theoretical factors, with 7–15 items up to the 11–13 that we have obtained, some of which were formed by only two or three items. In addition, we have been able to interpret only between seven and nine of these factors. Something like this has occurred in only one other study, which yielded nine factors in university students [41].

We could say that the factors representing positive mood states are more similar to the theoretical structure. Both have been clearly confirmed in various studies [36, 40], although most included only Vigor-Activity, not Friendliness [25, 34, 38, 39, 41].

In contrast, for the factors representing negative mood states, three trends could be observed. First, a super factor seems to emerge, consisting of a significantly larger number of items and measuring Tension-Anxiety, Depression-Dejection, and Anger-Hostility. Arguably, these illustrate the three most relevant emotions in chronic pain: anxiety, sadness, and anger. Other studies have produced similar results, hinting at a global factor of mood disorder [34]. A variant of this super factor, including Confusion-Bewilderment rather than Anger-Hostility, has been found in samples of psychiatric patients and smokers [40].

Second, there seems to be a conceptual overlap among Fatigue-Inertia, Confusion-Bewilderment, and Depression-Dejection, in a factor we have referred to as Despondency. This is congruent with the fact that, in many healthy samples such as university students and athletes, the Confusion-Bewilderment factor has not been confirmed [15, 37, 38].

Finally, very specific groupings appear, such as the factor that we have called Resentment, with Anger items, and others such as hedonism and determination, based on Vigor, Fatigue, Anger, and Confusion items (in the last three cases, with negative loads). Another splitting of factors—in this case, Depression and Tension—gives rise to two new factors, Worthlessness and Alertness, as has also been documented in university students [41]. Likewise, a different splitting of the Depression factor referred to as dysphoria has been found in university students and athletes [38].

Validity evidence based on the internal structure would have provided information about the construct validity of the test. As none of the structures fit, it was not possible to study any other kind of validity evidence (convergent or discriminant validity, for example) linked to other variables [63], as construct validity evidence is a prerequisite for this. Similarly, as there was no way to confirm whether a measure obtained with the test effectively represented the construct, comparing this measure with other variables would not have contributed to establishing validity.

For an understanding of the previous findings, it is necessary to clarify that the POMS aims to measure mood, not emotions. Mood is more durable than emotions and focuses exclusively on the phenomenological experience, exceeding the strictly emotional. Following Watson and Vaidya [63], there are two traditions in the conception and measurement of mood: one that focuses on global dimensions that reflect affective valence, such as positive affect vs negative affect [64], and another that aims to assess the content of specific moods, such as the POMS. In addition to the lack of consensus on a taxonomy of mood states, the main disadvantage of this second approach (i.e., the POMS) is the poor discriminant validity between the specific mood states. Therefore, it is much easier to confirm a general structure based on affective valence than on specific moods. Our results appear to confirm this, in keeping with the conclusions of some of the reference studies cited herein [38–41]. We also think that a chronic pain patient sample like ours could reflect an even greater fusion of mood states. In this regard, the fact that a diagnosis of fibromyalgia was exceedingly common in the sample could have influenced results, given evidence that the prevalence of alexithymia (difficulties in experiencing and handling emotions) is considerably higher among patients with fibromyalgia. In this regard, a recent study found a 47.9% prevalence rate of alexithymia among patients with fibromyalgia [65].

The semantic difficulty that seven items presented in the Spanish version merits special note, as it resulted in insufficient factor saturation. We believe that participants had trouble understanding the items “ready to fight,” “rebellious,” “carefree,” and “unworthy,” either because of problems contextualizing them or because of ambiguity. In other cases, there appeared to be understanding difficulties for the items “insightful” and “considerate,” which may be attributed to the low literacy level of the sample. This is not the first time that issues of this sort have been associated with a Spanish version of the POMS [15]. The case of the item “relaxed” poses a different issue altogether because, as other studies have documented, it has been loaded in Vigor instead of Tension [36]. This problem may have been exacerbated in our study by the fact that only one third of the sample had attended high school or college. In this regard, there is evidence of the need for instruments designed specifically for people with low educational attainment, who are also more likely to suffer from chronic pain [66].

We believe that the main limitation of our study was the lack of sample representativeness. As a voluntary convenience sample obtained essentially from public primary care centers, a very specific profile emerges: a middle-aged woman with low educational attainment, low to medium socioeconomic status, and a diagnosis of fibromyalgia with high chronicity. The three aspects most likely to limit the generalizability of the results include the lack of ethnic and cultural diversity in the sample (100% Spanish Caucasians), low educational attainment (two thirds had attended only elementary school, and some had no schooling at all), and the prevalence of a single diagnosis (fibromyalgia in 59% of the sample).

Another possible limitation is the fact that the same data set was used to develop Studies 1 and 2, though it is important to highlight that in Study 2, the data used for the EFAs differed from those used for the CFAs.

There are two potential alternatives to the full version of the POMS to measure emotional functioning in chronic pain. The first involves other mood measures, such as the Multiple Affect Adjective Checklist–Revised (MAACL-R), the Differential Emotions Scale (DES), and the Positive and Negative Affect Schedule–Expanded Form (PANAS-X). However, these bring their own risks and difficulties: excessive length and low discriminant validity in the MAACL-R and low reliability in the DES [63]. The proposal of the PANAS-X appears more robust and also offers the possibility of analyzing emotional functioning at the level of affective valence (positive and negative affect) or affective content (fear, sadness, guilt, hostility, joviality, self-assurance, attentiveness, shyness, fatigue, serenity, and surprise) [67]. The second alternative is to explore the factorial structure once the factor items have been refined [15, 37], a procedure that has been implemented in the shortened versions of the POMS [31, 38, 68] and that we propose as an objective for future studies.

## Supplementary Material

pnab127_Supplementary_Data

## Notes

Funding sources: Financial assistance was granted to the second author of the study (grant number BF1-18-04) by the Spanish Foundation of Pain and the Spanish Society of Pain. Financial assistance was also received through projects funded by the National Scientific and Technological Development Fund (FONDECYT), ANID, Government of Chile (ref. number 1190945), and the FEDER Operational Program 2014–2020 of the Andalusian government (ref. US-1263096), both granted to the third and fourth authors of the study.
