# Supplementary material for: Profile of Mood States Factor Structure Does Not Accurately Account for Patients with Chronic Pain
Source: Pain Med. 2021 Apr 2;22(11):2604–14. doi: 10.1093/pm/pnab127 (PMC8789763; doi:10.1093/pm/pnab127)
Supplement: pnab127_Supplementary_Data [file pnab127_supplementary_data.zip › Suppl 1.docx]

**Profile of Mood States (POMS). Spanish version by Arce, Andrade & Seoane (2000) from the original by McNair, Lorr & Droppleman (1971).**

La lista de palabras que aparece a continuación describe sentimientos que tienen las personas. Después de leer cada palabra fíjese en las cinco opciones posibles y elija la que mejor describa cómo se ha sentido usted últimamente rodeándola con un círculo según la siguiente escala:

**0 = Nada; 1 = Poco; 2 = Moderadamente; 3 = Bastante; 4 = Muchísimo**

| **1** | Amistoso | 0 | 1 | 2 | 3 | 4 | **33** | Resentido | 0 | 1 | 2 | 3 | 4 |
| --- | --- | --- | --- | --- | --- | --- | --- | --- | --- | --- | --- | --- | --- |
| **2** | Tenso | 0 | 1 | 2 | 3 | 4 | **34** | Nervioso | 0 | 1 | 2 | 3 | 4 |
| **3** | Enfadado | 0 | 1 | 2 | 3 | 4 | **35** | Solo | 0 | 1 | 2 | 3 | 4 |
| **4** | Rendido | 0 | 1 | 2 | 3 | 4 | **36** | Desdichado | 0 | 1 | 2 | 3 | 4 |
| **5** | Infeliz | 0 | 1 | 2 | 3 | 4 | **37** | Aturdido | 0 | 1 | 2 | 3 | 4 |
| **6** | Sensato | 0 | 1 | 2 | 3 | 4 | **38** | Alegre | 0 | 1 | 2 | 3 | 4 |
| **7** | Animado | 0 | 1 | 2 | 3 | 4 | **39** | Amargado | 0 | 1 | 2 | 3 | 4 |
| **8** | Confundido | 0 | 1 | 2 | 3 | 4 | **40** | Exhausto | 0 | 1 | 2 | 3 | 4 |
| **9** | Arrepentido | 0 | 1 | 2 | 3 | 4 | **41** | Ansioso | 0 | 1 | 2 | 3 | 4 |
| **10** | Agitado | 0 | 1 | 2 | 3 | 4 | **42** | Listo para pelear | 0 | 1 | 2 | 3 | 4 |
| **11** | Desatento | 0 | 1 | 2 | 3 | 4 | **43** | Afable | 0 | 1 | 2 | 3 | 4 |
| **12** | Malhumorado | 0 | 1 | 2 | 3 | 4 | **44** | Abatido | 0 | 1 | 2 | 3 | 4 |
| **13** | Considerado | 0 | 1 | 2 | 3 | 4 | **45** | Desesperado | 0 | 1 | 2 | 3 | 4 |
| **14** | Triste | 0 | 1 | 2 | 3 | 4 | **46** | Perezoso | 0 | 1 | 2 | 3 | 4 |
| **15** | Activo | 0 | 1 | 2 | 3 | 4 | **47** | Rebelde | 0 | 1 | 2 | 3 | 4 |
| **16** | Con nervios | 0 | 1 | 2 | 3 | 4 | **48** | Desvalido | 0 | 1 | 2 | 3 | 4 |
| **17** | Irritable | 0 | 1 | 2 | 3 | 4 | **49** | Cansado | 0 | 1 | 2 | 3 | 4 |
| **18** | Melancólico | 0 | 1 | 2 | 3 | 4 | **50** | Desorientado | 0 | 1 | 2 | 3 | 4 |
| **19** | Enérgico | 0 | 1 | 2 | 3 | 4 | **51** | Alerta | 0 | 1 | 2 | 3 | 4 |
| **20** | Descontrolado | 0 | 1 | 2 | 3 | 4 | **52** | Engañado | 0 | 1 | 2 | 3 | 4 |
| **21** | Desesperanzado | 0 | 1 | 2 | 3 | 4 | **53** | Furioso | 0 | 1 | 2 | 3 | 4 |
| **22** | Relajado | 0 | 1 | 2 | 3 | 4 | **54** | Eficiente | 0 | 1 | 2 | 3 | 4 |
| **23** | No merecedor | 0 | 1 | 2 | 3 | 4 | **55** | Confiado | 0 | 1 | 2 | 3 | 4 |
| **24** | Rencoroso | 0 | 1 | 2 | 3 | 4 | **56** | Lleno de alegría | 0 | 1 | 2 | 3 | 4 |
| **25** | Comprensivo | 0 | 1 | 2 | 3 | 4 | **57** | Con mal genio | 0 | 1 | 2 | 3 | 4 |
| **26** | Intranquilo | 0 | 1 | 2 | 3 | 4 | **58** | Inútil | 0 | 1 | 2 | 3 | 4 |
| **27** | Inquieto | 0 | 1 | 2 | 3 | 4 | **59** | Olvidadizo | 0 | 1 | 2 | 3 | 4 |
| **28** | Desconcentrado | 0 | 1 | 2 | 3 | 4 | **60** | Despreocupado | 0 | 1 | 2 | 3 | 4 |
| **29** | Fatigado | 0 | 1 | 2 | 3 | 4 | **61** | Aterrorizado | 0 | 1 | 2 | 3 | 4 |
| **30** | Servicial | 0 | 1 | 2 | 3 | 4 | **62** | Culpable | 0 | 1 | 2 | 3 | 4 |
| **31** | Molesto | 0 | 1 | 2 | 3 | 4 | **63** | Vigoroso | 0 | 1 | 2 | 3 | 4 |
| **32** | Desanimado | 0 | 1 | 2 | 3 | 4 | **64** | Inseguro | 0 | 1 | 2 | 3 | 4 |
|  |  |  |  |  |  |  | **65** | Agotado | 0 | 1 | 2 | 3 | 4 |
